# Flexible and directional fibre optic ultrasound transmitters using photostable dyes

**DOI:** 10.1364/OSAC.431444

**Published:** 2021-09-08

**Authors:** Richard J. Colchester, Callum D. Little, Erwin J. Alles, Adrien E. Desjardins

**Affiliations:** 1 Department of Medical Physics and Biomedical Engineering, University College London, Gower Street, London, WC1E 6BT, UK; 2 Wellcome/ESPRC Centre for Interventional and Surgical Sciences, 43-45 Foley Street, London, W1W 7TY, UK; 3 Department of Cardiovascular Medicine, Royal Free NHS Foundation Trust, London, UK

## Abstract

All-optical ultrasound transducers are well-suited for use in imaging during minimally invasive surgical procedures. This requires highly miniaturised and flexible devices. Here we present optical ultrasound transmitters for imaging applications based on modified optical fibre distal tips which allow for larger transmitter element sizes, whilst maintaining small diameter proximal optical fibre. Three optical ultrasound transmitter configurations were compared; a 400 µm core optical fibre, a 200 µm core optical fibre with a 400 µm core optical fibre distal tip, and a 200 µm core optical fibre with a 400 µm core capillary distal tip. All the transmitters used a polydimethylsiloxane-dye composite material for ultrasound generation. The material comprised a photostable infra-red absorbing dye to provide optical absorption for the ultrasound transduction. The generated ultrasound beam profile for the three transmitters was compared, demonstrating similar results, with lateral beam widths <1.7 mm at a depth of 10 mm. The composite material demonstrates a promising alternative to previously reported materials, generating ultrasound pressures exceeding 2 MPa, with corresponding bandwidths *ca.* 30 MHz. These highly flexible ultrasound transmitters can be readily incorporated into medical devices with small lateral dimensions.

## 1. Introduction

Highly miniaturised and flexible imaging devices can play transformative roles in guiding minimally invasive surgical procedures. All-optical ultrasound is an emerging paradigm that is well suited to these applications due to its promising properties. With this technique, ultrasound is both generated and received using light. Ultrasound generation occurs through the photoacoustic effect where excitation light delivered to an optical absorbing coating is absorbed leading to a temperature rise. This results in a pressure increase which propagates as an ultrasound wave [1]. Ultrasound reception relies on the use of interferometric methods where reflected ultrasound waves modulate the thickness of optically resonant structures. Accurate optical monitoring of these structures allows measurements of the reflected ultrasound [2–4]. Miniaturisation of all-optical ultrasound devices is possible through the use of optical fibres as substrates for the optical ultrasound generators and receivers [5–7]. These devices demonstrate broad bandwidths and high sensitivity, required for high resolution imaging in a minimally invasive context [5,6]. Further, all-optical ultrasound imaging devices can be made immune to electromagnetic interference, allowing for their use in conjunction with modalities such as magnetic resonance imaging or during radio-frequency ablation [8].

Several fibre optic ultrasound generators have been demonstrated, using a range of ultrasound generating materials and based on various sized optical fibres. For ultrasound generating coatings, composites comprising polydimethylsiloxane (PDMS) are commonly used due to the high thermal expansion coefficient of PDMS, which leads to high ultrasound pressure due to efficient conversion of the deposited optical energy to acoustic energy [9]. Many different materials have been incorporated into the PDMS to provide optical absorption, including metallic nanoparticles [9–11], carbon black [12,13], organic dyes [9], candle soot [14,15], carbon nanotubes [16–20], and graphene [21]. For imaging applications, studies have shown the advantages of using smaller optical fibres to provide a wide acoustic aperture when synthetic aperture image reconstruction for two- and three- dimensional imaging is feasible [5,18]. These fibres also provide high physical flexibility which is required in many surgical scenarios to reach challenging locations within the human body. However, for many surgical scenarios, the precise control or knowledge of device location required for synthetic aperture image reconstruction is not possible. For these situations, frequency filtering and the use of larger diameter optical fibre transmitters can provide increased resolution with images generated by concatenation of consecutive A-lines [6,22,23]. However, the use of larger diameter optical fibres comes with a trade-off in the physical flexibility of the device.

Here, we demonstrate two novel and highly mechanically flexible optical ultrasound generators which comprise a large diameter distal tip with a tapered proximal optical fibre section. This method maintains the overall device flexibility whilst providing a larger source dimensions for directional ultrasound generation. The two generators were compared with an equivalent sized non-tapered optical ultrasound generator. Further, the use of frequency filtering to further increase the directionality of the transmitted ultrasound was considered. Given the high mechanical flexibility of the proximal optical fibre, these optical ultrasound generators are highly suited for use in imaging during surgical scenarios which require devices to undergo tortuous pathways.

In this work three optical fibre ultrasound generators were compared: a monolithic 400 µm core diameter optical fibre, a 200 µm core optical fibre with a 400 µm core optical fibre distal tip, and a 200 µm core optical fibre with a 400 µm core capillary distal tip. For simplicity, these will be referred to as ‘monolithic’, ‘spliced’, and ‘capillary’ fibre ultrasound generators, respectively, throughout the manuscript.

## 2. Fabrication

### 2.1 Optical fabrication

**Fig. 1.**
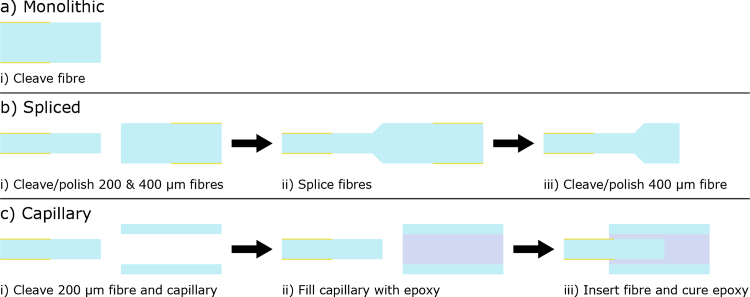
Schematic of fibre preparation for ultrasound generator fabrication. a) Monolithic fibre preparation: i) a 400 µm fibre is cleaved and polished. b) Spliced fibre preparation: i) 200 & 400 µm fibres are cleaved and polished, ii) the fibres are spliced together, iii) the 400 µm end is cleaved and polished. c) Capillary fibre preparation: i) 200 µm fibre and a glass capillary section are cleaved, ii) the capillary is filled with an optical epoxy, iii) the fibre is inserted into the capillary and the epoxy is cured.

The fabrication process for the optical fibre ultrasound transmitters is described in Fig. 1. For monolithic and spliced devices, 5 of each were fabricated, whilst for capillary devices 10 were fabricated. The monolithic fibre was prepared by removing a 1 cm length of the polyimide coating from the distal end of a 400 µm core diameter optical fibre (WF 400/440/470 P, CeramOptec, Germany), which had a minimum short term bend radius of 22 mm. The bare fibre was wiped clean using ethanol. Subsequently, the bare fibre section was manually cleaved 5 mm from the distal tip using a tungsten blade.

The spliced fibre comprised a 200 µm core diameter optical fibre (WF 200/210/235 P, CeramOptec, Germany), which had a minimum short term bend radius of 10.5 mm, spliced to a 400 µm core diameter optical fibre (WF 400/440/470 P, CeramOptec, Germany) (Fig. 1(b)). Both optical fibres were prepared by stripping a 1 cm length of the polyimide buffer coating from the distal tip of the optical fibre. Subsequently, the stripped section was cleaned using ethanol. The 200 µm core diameter optical fibre was cleaved using an motorised cleaver (CT- 101 , Fujikura, UK), whilst the 400 µm core diameter optical fibre was cleaved manually with a tungsten blade. Following cleaving, the end surface of the 400 µm core diameter optical fibre was polished flat to prepare the fibre for splicing. Polishing was not required for the 200 µm core diameter optical fibre due to the superior cleave quality. The prepared fibres were spliced together to form a single fibre with a step up in fibre core diameter from 200 to 400 µm (Fig. 1(b)). Following fusion splicing, the 400 µm fibre was cleaved *ca.* 5 mm distal to the splice point. The short 5 mm length was left to allow light divergence within the 400 µm section and to improve the ease of handling. The end surface of the 400 µm section spliced to the 200 µm fibre was subsequently polished to ensure a flat end surface.

The capillary fibre comprised a 200 µm core diameter optical fibre (WF 200/210/235 P, CeramOptec, Germany) inserted into a cylindrical glass capillary (Fig. 1(c)). The optical fibre was prepared by stripping a 1 cm length of the polyimide buffer coating from its distal tip. Subsequently, the stripped section was cleaned using ethanol and the fibre was cleaved. The capillary (ID: 400 µm, OD: 550 µm, CV 4055−10 , CM Scientific Ltd, UK) was prepared by cleaving a 5 mm long section using a tungsten blade. This length was chosen to improve the ease of handling and allow for light divergence within the capillary to increase the optical beam diameter. The capillary section was filled with a high refractive index epoxy (NOA 1665, Norland, USA) and the prepared optical fibre was inserted into the filled capillary until a 2 mm length of the optical fibre was inside the capillary. The epoxy was chosen due to its high refractive index of 1.665 , compared to *ca.* 1.5 for borosilicate glass. Thus, light guiding was expected within the capillary under the assumption the capillary walls were optically smooth. Finally, the epoxy was cured using a UV LED (wavelength: 365 nm, power: *ca.* 10 mW, M 365 FP 1 , Thorlabs, UK).

### 2.2 Composite coating

All ultrasound generator types were coated using the same composite coating and dip coating protocol. A dye-PDMS dip coating solution was prepared. Firstly, 10 mg of near-infrared absorbing dye (Epolight 9837, Epolin, USA) was manually stirred in 0.5 ml xylene until a homogeneous solution was achieved. This dye was chosen due to the good photostability and high optical absorption at 1064 nm, as per the manufacturer’s specifications. Subsequently, 250 mg of PDMS (MED-1000, Polymer Systems Technology, UK) was added to the solution and manually stirred until homogeneous. To coat the prepared fibre optic ultrasound transmitters, the prepared devices were manually dipped in the dye-PDMS solution, then removed and stored with the coated surface facing up. The coatings were left for 24 hours in ambient conditions to cure.

## 3. Characterisation

Prior to ultrasound characterisation, the devices coated with the dye-PDMS composite were examined visually using a stereo-microscope and the optical absorption of the dye-PDMS composite at a wavelength of 1064 nm was measured. The ultrasound generated by the devices was characterised using a 200 µm needle hydrophone (Precision Acoustics, UK) with a calibration range 1−30 MHz. The device under test was coupled to a Q-switched Nd:YAG laser (Spot- 10 - 500 - 1064 , Elforlight, UK) for ultrasound excitation. The laser had a pulse width of 2 ns, a repetition rate of 100 Hz, and a pulse energy of 20.1 µJ. The ultrasound field was scanned by raster scanning the hydrophone over a 2 D grid orthogonal to the longitudinal axis of the optical fibre. The grid was 3×3 mm with a uniform step size of 50 µm and was positioned at an axial distance of 1.5 mm from the distal tip of the device under test. Further, the photostability of the coatings was tested by measuring the ultrasound output before and after continuous laser excitation for 2 hours, using the same laser parameters as used for the ultrasound field scans.

The measured ultrasound field scan was numerically back- and forward-propagated to determine the divergence of the ultrasound beam. For this, the angular spectrum approach [24] was applied to propagate the ultrasound pressure field to axial distances ranging between 0 and 10 mm in steps of 1.25 mm. Further, a second propagation was carried out for each device using a high-pass filter with a cut-off frequency of 20 MHz to improve the directionality of the ultrasound beam.

## 4. Results

**Fig. 2.**
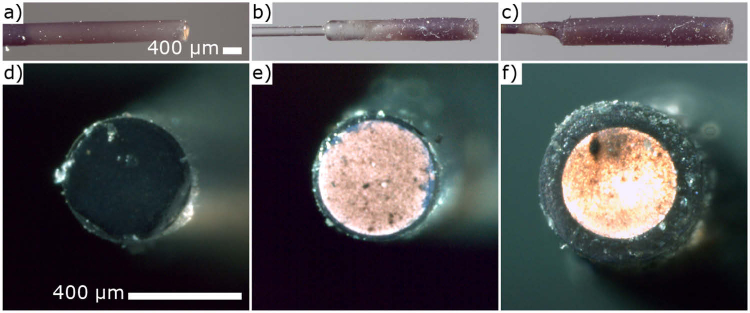
Side on microscope images of a) Monolithic, b) Spliced, and c) Capillary fibre transmitters (Scale same for a) - c)). End on microscope images of d) Monolithic (with no through illumination), e) Spliced (with through illumination), and f) Capillary (with through illumination) fibre transmitters (Scale same for d) - f)).

The dip coating process produced smooth even coatings over all three types of device (Fig. 2) and the optical absorption at 1064 nm was >95% for all coatings. Observation of the coating thickness using stereomicroscope revealed coating thicknesses <20 µm for all the devices. For the monolithic and spliced devices the resulting ultrasound fields were consistent across all the devices made, with a variation in absolute pressure and bandwidth between devices <15% . However, for the spliced devices, half of the fabricated devices generated ultrasound pressures <85% of the best capillary device and were thereby deemed unacceptable. For all devices, no change in the ultrasound output was found after continuous excitation for a 2 hour period.

**Fig. 3.**
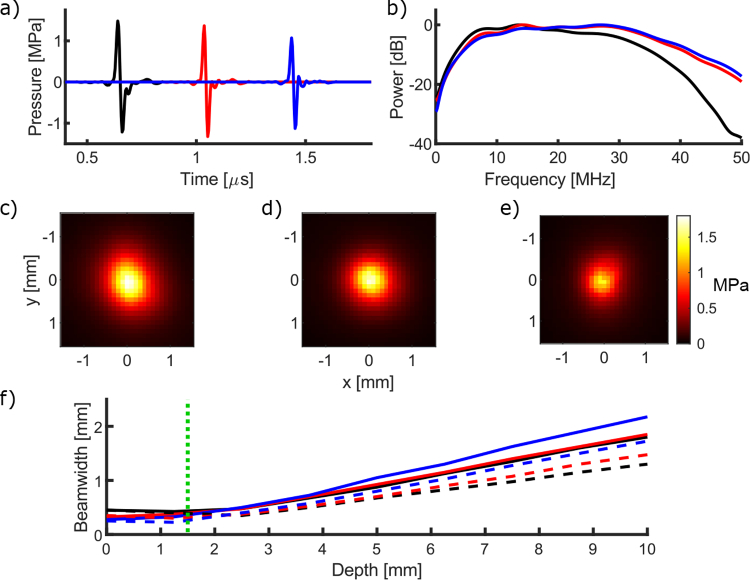
a) Ultrasound time-series as measured at 1.5 mm from the devices; monolithic (black line, shifted −0.4 µs for display), spliced (red line, not shifted), and capillary (blue line, shifted +0.4 µs for display). b) Corresponding ultrasound spectra from the devices normalised to 0 . c), d), e) Ultrasound field scans for the monolithic, spliced, and capillary devices, respectively, as measured at 1.5 mm from the device, plotted with the same colour scale for comparison. f) Full-width half-maximum ultrasound beamwidth for the devices (green dotted line shows the measurement distance), solid line: full power spectrum, dashed line: with >20 MHz high pass filtering.

The characteristics of the generated ultrasound are summarised in Table 1. The results quoted are for the best performing of each of the devices fabricated, however, they are representative of all of the acceptable devices. The peak-to-peak ultrasound pressure in the centre of the beam at a distance of 1.5 mm was similar for the three device types, with values of 2.70 , 2.69 , and 2.20 MPa, for the monolithic, spliced, and capillary devices, respectively (Fig. 3(a)). The corresponding −6 dB bandwidths for the devices were 28.5 , 31.7 , and 32.6 MHz, respectively (Fig. 3(b)).

**Table 1.** Generated ultrasound characteristics for the three types of device: monolithic, spliced, and capillary. Measured ultrasound pressure and bandwidth where recorded at a distance of 1.5 mm from the device.

**Generator Type**	**Pk-Pk Pressure**	**−6 dB Bandwidth**	**Beamwidth @ 10 mm [mm]**	
	**[MPa]**	**[MHz]**	**> 1 MHz**	**> 20 MHz**

*Monolithic*	2.70	28.5	1.8	1.3
*Spliced*	2.69	31.7	1.9	1.5
*Capillary*	2.20	32.6	2.2	1.7

The field measurements showed that the ultrasound generated by all three device types was circularly symmetric (Fig. 3(c), (d), (e)). The full width half maximum beamwidth was similar with increasing axial depth for the device types, however, the monolithic device had the narrowest beam width at a depth of 10 mm with a value of 1.8 mm (Fig. 3(f)). The capillary device had the largest with a value of 2.2 mm. When high-pass frequency filtering was applied the beamwidth decreased for all devices, with values of 1.7 , 1.5 , 1.3 mm for the capillary, spliced, and monolithic devices, respectively. These correspond to angular divergences of $9.3^{\circ }$ , $7.9^{\circ }$ and $6.4^{\circ }$ for the capillary, spliced, and monolithic devices, respectively.

## 5. Discussion and conclusion

Here, we presented two methods for fabricating highly flexible optical ultrasound generators with a small diameter optical fibre and a larger distal section for ultrasound generation. One method comprised splicing a 400 µm core diameter optical fibre section onto the end of a 200 µm core diameter optical fibre, whilst the other comprised an epoxy filled glass capillary (ID: 400 µm, OD: 550 µm) affixed to the distal end of a 200 µm optical fibre. The use of a smaller diameter proximal fibre provided a low minimum bend radius of 10.5 mm, making them suitable for use in complex and tortuous anatomies. Both the fabricated devices were compared to an ultrasound generator based on a monolithic cleaved 400 µm core diameter optical fibre. All devices were coated with a dye-PDMS optical ultrasound generating coating. The combination of highly flexible devices with directional ultrasound generation makes these devices ideally suited to minimally-invasive imaging applications where devices must undergo tortuous pathways and provide real-time imaging.

The coatings fabricated exhibited high optical absorption and inspection of the coatings showed that they formed uniform layers across the tips of all devices and were <20 µm thick. The ultrasound pressures generated by all devices was comparable to those previously used for *in vivo* imaging [6]. Further, the corresponding −6 dB bandwidth for both the flexible devices (spliced and capillary) exceeded 30 MHz, crucial for providing high axial resolution when imaging [5,6,9]. Small variations in the bandwidth between the devices are likely due to small inconsistencies in the coating due to the manual nature of the dip coating process. Observations suggest that the photostability of the dye-PDMS coatings was excellent. No change in acoustical performance was observed during normal lab usage over a timescale of *ca.* 2 hours whilst submerged in water. This suggests the devices are suitable for minimally invasive surgical procedures which typically require imaging over periods of less than one hour, depending on the clinical context.

For all devices, the full width half maximum beamwidth calculated at 10 mm from the device was *ca.* 2 mm. Whilst the smallest beamwidth was found for the monolithic device, the spliced device exhibited a beamwidth 0.1 mm larger at 10 mm. The larger beamwidth for the capillary device may be due to slight misalignment in the manual connectorisation of the capillary and optical fibre using epoxy. This could result in a potentially different distribution of the optical energy on the coating as compared with the other device types, resulting in a different beamwidth. Using frequency filtering to remove the more divergent lower frequencies, the beamwidth for all devices at 10 mm was reduced to <2 mm. The high directionality achieved by these devices, which was $<9.3^{\circ }$ for all devices with the application of high-pass filtering (cut-off frequency: 20 MHz), suggest that these sources are well-suited to M-mode or sweep imaging, where the lateral imaging resolution is dependent on the beamwidth. The divergence angles of the ultrasound beams in this study were comparable to the value of $7^{\circ }$ achieved by Colchester *et al.* using a 600 µm core diameter optical fibre [21]. The similarity of these divergence angles is likely due to the combination of a wider ultrasound bandwidth generated by the devices fabricated in this study and transmitting element size.

Each of the two fabrication methods has its advantages. Whilst the spliced device outperformed the capillary device in terms of the performance consistency across different devices and of the beam divergence, the fabrication process required polishing and preparation steps to facilitate optical fibre splicing. During this process there was a risk of damaging the 400 µm fibre section. Devices had to be processed in series and each step was time consuming and required specialised equipment, including an optical fibre splicer and fibre polishing facilities. The failure rate during fabrication was *ca.* 50% due to the delicate nature of the fibre splicing process. Conversely, the capillary devices did not require specialised equipment and could be made using off-shelf epoxy and a low cost UV light source. Additionally, the process allowed for parallel fabrication of several devices in one batch, making it highly scalable for production of large numbers of devices. However, the device performance for the final devices less consistent than it was with spliced devices, with *ca.* 50% of devices not meeting the acceptability criterion of generating >85% of the absolute pressure generated by the best performing capillary fibre. For capillary devices it was possible to fabricate 20 devices within a 3 hour period, from start to finish, and this number could easily be scaled. Whilst for spliced devices, a similar number of devices would require 20 hours.

The devices presented here overcome the minimum bend radius limitation of previous devices ( >22 mm) [6,21], and reduce this to a minimum bend radius of 10.5 mm. In this study a 200 µm core diameter optical fibre was used for the proximal section to match the output fibre from the excitation laser. However, this diameter could be further reduced if the laser was coupled into a smaller optical fibre, leading to a further reduction in the minimum bend radius. For example, a 105 µm core diameter optical fibre could provide a minimum bend radius of <6 mm. Previous devices have used larger optical fibres to provide larger ultrasound generating apertures, up to 600 µm [21] or much larger concave structures [22], however, due to the high ultrasound bandwidth achieved in this study, comparable directionalities were achieved. The methods presented in this study could be adapted to incorporate wider ultrasound generating apertures, which may lead to narrower beamwidths [21]. However, it should be noted that the reduction in beam divergence diminishes for increasing aperture diameter. So whilst increasing the aperture diameter can have a large effect for small fibres, the effect is diminished for larger aperture fibres. Further, the maximum device size will depend on the space constraints of the application. Larger apertures could be achieved either by splicing to larger core fibre, or using larger diameter capillaries. This might require a stepped process, with splicing gradually increasing core diameter fibres sequentially, or similar with capillaries, to provide device stability.

The results presented in this study demonstrate two methods for fabricating flexible and highly directional optical ultrasound transmitters. The generated ultrasound characteristics suggest that the directional sources are well-suited for high-resolution M-mode imaging. The directionality achieved here exceeds that of a device used to provide M-mode imaging in a previous *in vivo* study [6] and the beamwidth at 10 mm is comparable to that of a pencil beam all-optical ultrasound imaging device used to image a swine aorta *ex vivo* [22]. Both methods could be readily scaled using industrial processes and lend themselves to inexpensive fabrication. High collimation was achieved whilst more than halving the bend radius and comparison to a monolithic cleaved fibre demonstrated comparable performance. Furthermore, the composite coating comprising PDMS and an off-the-shelf dye was shown as a viable alternative to other commonly used materials. Use of an off-the-shelf dye facilitated a straight-forward manual mixing fabrication method without the need for specialised equipment. Further, as per the manufacturers specifications, the dye is expected to exhibit a low optical absorption across visible wavelengths. This may be explored in future studies to enable the addition of complementary imaging and therapeutic modalities to the devices, as with previous studies [9]. The presented flexible and directional optical ultrasound devices are hence well-suited to minimally invasive interventional applications.

## Data Availability

Data underlying the results presented in this paper are not publicly available at this time but may be obtained from the authors upon reasonable request.
